# Including metabolite concentrations into flux balance analysis: thermodynamic realizability as a constraint on flux distributions in metabolic networks

**DOI:** 10.1186/1752-0509-1-23

**Published:** 2007-06-01

**Authors:** Andreas Hoppe, Sabrina Hoffmann, Hermann-Georg Holzhütter

**Affiliations:** 1 Charité – University Medicine Berlin, Institute for Biochemistry, Universitätsmedizin Berlin, Monbijoustr. 2, 10117 Berlin, Germany

## Abstract

**Background:**

In recent years, constrained optimization – usually referred to as flux balance analysis (FBA) – has become a widely applied method for the computation of stationary fluxes in large-scale metabolic networks. The striking advantage of FBA as compared to kinetic modeling is that it basically requires only knowledge of the stoichiometry of the network. On the other hand, results of FBA are to a large degree hypothetical because the method relies on plausible but hardly provable optimality principles that are thought to govern metabolic flux distributions.

**Results:**

To augment the reliability of FBA-based flux calculations we propose an additional side constraint which assures thermodynamic realizability, i.e. that the flux directions are consistent with the corresponding changes of Gibb's free energies. The latter depend on metabolite levels for which plausible ranges can be inferred from experimental data. Computationally, our method results in the solution of a mixed integer linear optimization problem with quadratic scoring function. An optimal flux distribution together with a metabolite profile is determined which assures thermodynamic realizability with minimal deviations of metabolite levels from their expected values. We applied our novel approach to two exemplary metabolic networks of different complexity, the metabolic core network of erythrocytes (30 reactions) and the metabolic network iJR904 of *Escherichia coli *(931 reactions). Our calculations show that increasing network complexity entails increasing sensitivity of predicted flux distributions to variations of standard Gibb's free energy changes and metabolite concentration ranges. We demonstrate the usefulness of our method for assessing critical concentrations of external metabolites preventing attainment of a metabolic steady state.

**Conclusion:**

Our method incorporates the thermodynamic link between flux directions and metabolite concentrations into a practical computational algorithm. The weakness of conventional FBA to rely on intuitive assumptions about the reversibility of biochemical reactions is overcome. This enables the computation of reliable flux distributions even under extreme conditions of the network (e.g. enzyme inhibition, depletion of substrates or accumulation of end products) where metabolite concentrations may be drastically altered.

## Background

Sequencing the whole genomes in conjunction with high-throughput analyses of mRNA, protein and metabolite profiles [1] has paved the way for a fast reconstruction of metabolic networks [2]. For a quantitative assessment of metabolic fluxes, Palsson and co-workers have developed a theoretical approach commonly referred to as flux balance analysis (FBA) [3]. This method relies on the hypothesis that the most likely distribution of stationary fluxes in the network has to be optimal with respect to a feasible optimization criterion linking the fluxes with cellular functions. In most applications of FBA the fluxes have been determined to maximize a specific network output as the production of biomass [4-6]) or the production of ethanol in yeast [7]. Whereas the maximization of biomass production appears to be a reasonable objective of the cellular metabolism of rapidly growing and replicating primitive cells such as bacteria, the flux distribution in complex eukaryotic cells is governed by a larger variety of cellular functions that have to be met simultaneously. Therefore, the principle of flux-minimization was proposed as a more general optimization criterion for FBA [8-10]. The extension of FBA outlined in this paper will be tested by choosing as flux objective both the maximization of biomass and the minimization of internal fluxes at given output fluxes. Flux distributions predicted by FBA are hypothetical because they depend essentially upon the choice of the flux evaluation criterion used. Therefore, to increase the reliability of FBA results, one has to seek for strategies to include additional biochemical knowledge into FBA. One way is to include measured flux rates as further side constraints. However, flux measurements – except for exchange reactions that deliver metabolites into the external space – are still difficult and costly to perform as they require determining labeled isotopomers in a time-dependent manner [11-13]. Another possibility to increase the credibility of flux balance calculations is to include some basic thermodynamics of the reactions and transport processes constituting the network. The thermodynamic consensus rule dictates that a positive net flux through a reaction implies a negative corresponding change of the Gibb's free reaction energy and vice versa. Based on this fundamental criterion one may check whether given flux directions conflict with known Gibb's free energy changes. This allows to identify putative regulatory sites in the network [14,15] or to decide on the reversibility/irreversibility of reactions [16-20]. However, regarding flux distributions predicted by constrained optimization methods as FBA it is desirable to judge their feasibility not only post-hoc but to include thermodynamic constraints on flux directionalities directly into their calculation [21-23]. In our previous work [8,9] this was accomplished by weighting negative (backward) fluxes with the thermodynamic equilibrium constant of the reaction. The rationale behind this empirical weighting procedure is to impede reversing the direction of a reaction (such that the change of Gibb's free energy has the opposite sign than under standard conditions) with increasing value of the thermodynamic equilibrium constant. However, this way of mixing the costs for the maintenance of metabolic fluxes with the thermodynamic 'costs' for reversing the direction of a reaction in one and the same objective function is questionable for two reasons. First, the concentrations of metabolites in a cell differ significantly from 1 M so that the actual free energy changes of biochemical reactions may considerably differ from their standard values. Second, increasing or decreasing the concentration of the reactants to an extent that enables reversal of the flux direction might occur in the cell by regulations that does not cause much real 'costs' in terms of the production of more enzyme and of used external resources. One way to overcome this shortcoming of our previous approach [8,9] is to incorporate fulfillment of the thermodynamic consensus rule as additional side constraint into the calculation of the flux distribution. Such an approach was recently outlined by Henry et al. [23]. These authors studied the range of metabolite concentrations that is still compatible with a thermodynamically feasible flux distribution in a genome-scale network of *E. coli *under conditions of optimal bacterial growth. Here we go one step further to include information on metabolite concentrations directly into the calculation of the flux distribution. Our algorithm considers the optimization of two different objectives: On one hand a functionally optimal and thermodynamically feasible flux distribution is demanded an on the other hand the calculated metabolite concentrations are required to deviate as little as possible from set-point values prescribed on the basis of biochemical knowledge. In the following we outline the method and provide applications to two different metabolic networks: (i) the energy- and redox metabolism of red blood cells for which a detailed kinetic model has been established [24] thus allowing to check the feasibility of our method and (ii) the large-scale genome-based metabolic network of *Escherichia coli *iJR904 [25] which has already been subjected to FBA in several studies [23,26,27].

## Results

### Algorithm

#### Thermodynamic constraints

The directionality of the net flux of a chemical reaction and the change of Gibb's free energy are related to each other by the consensus rule

sgn(*v*) = -sgn (ΔG_r_),

where 'sgn()' is the sign function, ΔG_r _denotes the change of Gibb's energy of the reaction and *v *is the net flux (rate) through the reaction. Actual changes of Gibb's energy can be calculated from changes of standard Gibb's energy (where each reactant has a concentration of 1 M) according to

ΔGr=ΔGr0+RT∑M∈Pln⁡[M]−RT∑M∈Sln⁡[M],
 MathType@MTEF@5@5@+=feaafiart1ev1aaatCvAUfKttLearuWrP9MDH5MBPbIqV92AaeXatLxBI9gBaebbnrfifHhDYfgasaacH8akY=wiFfYdH8Gipec8Eeeu0xXdbba9frFj0=OqFfea0dXdd9vqai=hGuQ8kuc9pgc9s8qqaq=dirpe0xb9q8qiLsFr0=vr0=vr0dc8meaabaqaciaacaGaaeqabaqabeGadaaakeaacqqHuoarcqqGhbWrdaWgaaWcbaGaeeOCaihabeaakiabg2da9iabfs5aejabbEeahnaaDaaaleaacqqGYbGCaeaacqaIWaamaaGccqGHRaWkcqqGsbGucqWGubavdaaeqbqaaiGbcYgaSjabc6gaUjabcUfaBjabd2eanjabc2faDjabgkHiTiabbkfasjabdsfaubWcbaGaemyta0KaeyicI4SaeeiuaafabeqdcqGHris5aOWaaabuaeaacyGGSbaBcqGGUbGBcqGGBbWwcqWGnbqtcqGGDbqxaSqaaiabd2eanjabgIGiolabbofatbqab0GaeyyeIuoakiabcYcaSaaa@570E@

where [*M*] is the active concentration (activity) of the metabolite *M*, S and P denote the sets of substrates and products of the reaction, respectively. R is the universal gas constant and *T *is the absolute temperature. The change of the standard Gibb's energy is related to the equilibrium constant *K*_eq _of the reaction by

ΔGr0=−RTln⁡Keq.
 MathType@MTEF@5@5@+=feaafiart1ev1aaatCvAUfKttLearuWrP9MDH5MBPbIqV92AaeXatLxBI9gBaebbnrfifHhDYfgasaacH8akY=wiFfYdH8Gipec8Eeeu0xXdbba9frFj0=OqFfea0dXdd9vqai=hGuQ8kuc9pgc9s8qqaq=dirpe0xb9q8qiLsFr0=vr0=vr0dc8meaabaqaciaacaGaaeqabaqabeGadaaakeaacqqHuoarcqqGhbWrdaqhaaWcbaGaeeOCaihabaGaeGimaadaaOGaeyypa0JaeyOeI0IaeeOuaiLaemivaqLagiiBaWMaeiOBa4Maem4saS0aaSbaaSqaaiabbwgaLjabbghaXbqabaGccqGGUaGlaaa@3DBF@

It has to be noted that standard Gibb's energy changes depend on temperature, pH value, and ion strength and thus may significantly differ from those determined under specific in vitro conditions. For a metabolic network, (eq. 2) reads in vector notation ΔGr¯RT=ΔGr0¯RT+SC
 MathType@MTEF@5@5@+=feaafiart1ev1aaatCvAUfKttLearuWrP9MDH5MBPbIqV92AaeXatLxBI9gBaebbnrfifHhDYfgasaacH8akY=wiFfYdH8Gipec8Eeeu0xXdbba9frFj0=OqFfea0dXdd9vqai=hGuQ8kuc9pgc9s8qqaq=dirpe0xb9q8qiLsFr0=vr0=vr0dc8meaabaqaciaacaGaaeqabaqabeGadaaakeaadaWcaaqaamaanaaabaGaeuiLdqKaee4raC0aaSbaaSqaaiabbkhaYbqabaaaaaGcbaGaeeOuaiLaemivaqfaaiabg2da9maalaaabaWaa0aaaeaacqqHuoarcqqGhbWrdaqhaaWcbaGaeeOCaihabaGaeeimaadaaaaaaOqaaiabbkfasjabdsfaubaacqGHRaWkieqacqWFtbWucqqGdbWqaaa@3EF0@, where ΔGr¯
 MathType@MTEF@5@5@+=feaafiart1ev1aaatCvAUfKttLearuWrP9MDH5MBPbIqV92AaeXatLxBI9gBaebbnrfifHhDYfgasaacH8akY=wiFfYdH8Gipec8Eeeu0xXdbba9frFj0=OqFfea0dXdd9vqai=hGuQ8kuc9pgc9s8qqaq=dirpe0xb9q8qiLsFr0=vr0=vr0dc8meaabaqaciaacaGaaeqabaqabeGadaaakeaadaqdaaqaaiabfs5aejabbEeahnaaBaaaleaacqqGYbGCaeqaaaaaaaa@30CF@ is the column vector of the ΔG_r _values for all reactions of the network, ΔGr0¯
 MathType@MTEF@5@5@+=feaafiart1ev1aaatCvAUfKttLearuWrP9MDH5MBPbIqV92AaeXatLxBI9gBaebbnrfifHhDYfgasaacH8akY=wiFfYdH8Gipec8Eeeu0xXdbba9frFj0=OqFfea0dXdd9vqai=hGuQ8kuc9pgc9s8qqaq=dirpe0xb9q8qiLsFr0=vr0=vr0dc8meaabaqaciaacaGaaeqabaqabeGadaaakeaadaqdaaqaaiabfs5aejabbEeahnaaDaaaleaacqqGYbGCaeaacqqGWaamaaaaaaaa@31B7@ is the column vector of the ΔGr0
 MathType@MTEF@5@5@+=feaafiart1ev1aaatCvAUfKttLearuWrP9MDH5MBPbIqV92AaeXatLxBI9gBaebbnrfifHhDYfgasaacH8akY=wiFfYdH8Gipec8Eeeu0xXdbba9frFj0=OqFfea0dXdd9vqai=hGuQ8kuc9pgc9s8qqaq=dirpe0xb9q8qiLsFr0=vr0=vr0dc8meaabaqaciaacaGaaeqabaqabeGadaaakeaacqqHuoarcqqGhbWrdaqhaaWcbaGaeeOCaihabaGaeeimaadaaaaa@31A6@ values, C is the column vector of the natural logarithms of the active metabolite concentrations. (Concentrations are assumed to be strictly positive.) **S **is the stoichiometric matrix of the system, where rows refer to reactions and columns refer to metabolites. A positive or negative matrix element of **S **represents the stoichiometric coefficient with which the metabolite indicated by the column number appears as a product or substrate of the reaction indicated by the row number. Changes of the standard Gibb's energies of reactions can be additively composed of changes of the standard Gibb's energies of the formation of their reactants [28]:

ΔGr0¯=SΔGf¯.
 MathType@MTEF@5@5@+=feaafiart1ev1aaatCvAUfKttLearuWrP9MDH5MBPbIqV92AaeXatLxBI9gBaebbnrfifHhDYfgasaacH8akY=wiFfYdH8Gipec8Eeeu0xXdbba9frFj0=OqFfea0dXdd9vqai=hGuQ8kuc9pgc9s8qqaq=dirpe0xb9q8qiLsFr0=vr0=vr0dc8meaabaqaciaacaGaaeqabaqabeGadaaakeaadaqdaaqaaiabfs5aejabbEeahnaaDaaaleaacqqGYbGCaeaacqqGWaamaaaaaOGaeyypa0dcbeGae83uam1aa0aaaeaacqqHuoarcqqGhbWrdaWgaaWcbaGaeeOzaygabeaaaaGccqGGUaGlaaa@38F5@

Owing to the first law of thermodynamics the values of the standard Gibb's free energy changes are not independent from each other but have to obey the principle of micro-reversibility dictating the sum of standard free energy values in a closed system to be zero. In several flux balance studies [16,17,29-32] this criterion has been referred to as generalization of Kirchhoff's loop law which [see Additional file 3]. The problem is that experimentally determined values for the changes of standard Gibb's energies are not consistent with the principle of micro-reversibility per se because of experimental errors. Therefore, we add correction terms (forming the vector E) to all observed values of standard Gibb's energy changes and determine minimal corrections necessary to assure the principle of micro-reversibility. The corresponding optimization problem reads

Minimize‖E‖SubjecttoΔGr0¯∗=SΔGf¯∗+E,
 MathType@MTEF@5@5@+=feaafiart1ev1aaatCvAUfKttLearuWrP9MDH5MBPbIqV92AaeXatLxBI9gBaebbnrfifHhDYfgasaacH8akY=wiFfYdH8Gipec8Eeeu0xXdbba9frFj0=OqFfea0dXdd9vqai=hGuQ8kuc9pgc9s8qqaq=dirpe0xb9q8qiLsFr0=vr0=vr0dc8meaabaqaciaacaGaaeqabaqabeGadaaakeaafaqaaeGacaaabaacbeGae8xta0Kae8xAaKMae8NBa4Mae8xAaKMae8xBa0Mae8xAaKMae8NEaONae8xzaugabaWaauWaaeaacqqGfbqraiaawMa7caGLkWoaaeaacqWFtbWucqWF1bqDcqWFIbGycqWFQbGAcqWFLbqzcqWFJbWycqWF0baDcqWFGaaicqWF0baDcqWFVbWBaeaadaqdaaqaaiabfs5aejabbEeahnaaDaaaleaacqqGYbGCaeaacqqGWaamaaaaaOWaaWbaaSqabeaacqGHxiIkaaGccqGH9aqpcqWFtbWudaqdaaqaaiabfs5aejabbEeahnaaBaaaleaacqqGMbGzaeqaaaaakmaaCaaaleqabaGaey4fIOcaaOGaey4kaSIaeeyrauKaeiilaWcaaaaa@58FB@

where ||E|| is the 2-norm of the vector E, and ΔGf¯∗
 MathType@MTEF@5@5@+=feaafiart1ev1aaatCvAUfKttLearuWrP9MDH5MBPbIqV92AaeXatLxBI9gBaebbnrfifHhDYfgasaacH8akY=wiFfYdH8Gipec8Eeeu0xXdbba9frFj0=OqFfea0dXdd9vqai=hGuQ8kuc9pgc9s8qqaq=dirpe0xb9q8qiLsFr0=vr0=vr0dc8meaabaqaciaacaGaaeqabaqabeGadaaakeaadaqdaaqaaiabfs5aejabbEeahnaaBaaaleaacqqGMbGzaeqaaaaakmaaCaaaleqabaGaey4fIOcaaaaa@31DD@ are hypothetic Gibb's free energy changes of formation. ΔGr0¯∗−E
 MathType@MTEF@5@5@+=feaafiart1ev1aaatCvAUfKttLearuWrP9MDH5MBPbIqV92AaeXatLxBI9gBaebbnrfifHhDYfgasaacH8akY=wiFfYdH8Gipec8Eeeu0xXdbba9frFj0=OqFfea0dXdd9vqai=hGuQ8kuc9pgc9s8qqaq=dirpe0xb9q8qiLsFr0=vr0=vr0dc8meaabaqaciaacaGaaeqabaqabeGadaaakeaadaqdaaqaaiabfs5aejabbEeahnaaDaaaleaacqqGYbGCaeaacqqGWaamaaaaaOWaaWbaaSqabeaacqGHxiIkaaGccqGHsislcqqGfbqraaa@34E5@ is then used as the modified vector of standard Gibb's energy changes fulfilling the condition of micro-reversibility.

#### Constraints on metabolite concentrations

In case that the metabolite concentrations might assume arbitrary non-negative values it would be always possible to let a chemical reaction proceed in either forward or backward direction. Hence, including information on metabolite concentrations as additional constraints in FBA makes only sense if the concentration of the metabolites can be restricted to a feasible range. If the concentration of a metabolite is known, we use this value as set-point which should be approximated as best as possible by the calculated metabolite concentration. Thus, we add the term

∑m∈W(cm−sm)2
 MathType@MTEF@5@5@+=feaafiart1ev1aaatCvAUfKttLearuWrP9MDH5MBPbIqV92AaeXatLxBI9gBaebbnrfifHhDYfgasaacH8akY=wiFfYdH8Gipec8Eeeu0xXdbba9frFj0=OqFfea0dXdd9vqai=hGuQ8kuc9pgc9s8qqaq=dirpe0xb9q8qiLsFr0=vr0=vr0dc8meaabaqaciaacaGaaeqabaqabeGadaaakeaadaaeqbqaaiabcIcaOiabdogaJnaaBaaaleaacqWGTbqBaeqaaOGaeyOeI0Iaem4Cam3aaSbaaSqaaiabd2gaTbqabaGccqGGPaqkdaahaaWcbeqaaiabikdaYaaaaeaacqWGTbqBcqGHiiIZcqqGxbWvaeqaniabggHiLdaaaa@3C8E@

to the objective function where W denotes the set of metabolites *m *for which a set-point (logarithmic) concentration value *s*_*m *_is available and *c*_*m *_is the component of C related to metabolite *m*. If the concentration of a metabolite is not exactly known but can be restricted to a narrower concentration range based on metabolite profiling (for *E. coli *such a profile has been summarized by Kümmel et al. [14]) we use this information to define so-called soft concentration bounds denoted by *c*_low _and *c*_high_. In case that such physiologically feasible concentration range has not been reported yet we set the lower and upper soft bound close to the minimum and maximum of all known cellular metabolite concentrations. However, it may happen that a non-trivial flux distribution can only be found if the concentration of some metabolites drops off the range defined by the soft bounds. This may be due to the improper choice of soft bounds for some metabolites resulting from large experimental errors in the determination of cellular metabolite concentrations or the (unknown) binding of metabolites to macromolecular structures lowering their effective free concentrations inside the cell. Therefore, concentrations lying outside the range of the soft bounds are allowed in our algorithm but are penalized in the optimization criterion:

s(c)={clow−cif c<clow0if clow≤c≤chighc−chighif c>chigh
 MathType@MTEF@5@5@+=feaafiart1ev1aaatCvAUfKttLearuWrP9MDH5MBPbIqV92AaeXatLxBI9gBaebbnrfifHhDYfgasaacH8akY=wiFfYdH8Gipec8Eeeu0xXdbba9frFj0=OqFfea0dXdd9vqai=hGuQ8kuc9pgc9s8qqaq=dirpe0xb9q8qiLsFr0=vr0=vr0dc8meaabaqaciaacaGaaeqabaqabeGadaaakeaacqWGZbWCcqGGOaakcqWGJbWycqGGPaqkcqGH9aqpdaGabeqaauaabaqadiaaaeaacqWGJbWydaWgaaWcbaGaeeiBaWMaee4Ba8Maee4DaChabeaakiabgkHiTiabdogaJbqaaiabbMgaPjabbAgaMjabbccaGiabdogaJjabgYda8iabdogaJnaaBaaaleaacqqGSbaBcqqGVbWBcqqG3bWDaeqaaaGcbaGaeGimaadabaGaeeyAaKMaeeOzayMaeeiiaaIaem4yam2aaSbaaSqaaiabbYgaSjabb+gaVjabbEha3bqabaGccqGHKjYOcqWGJbWycqGHKjYOcqWGJbWydaWgaaWcbaGaeeiAaGMaeeyAaKMaee4zaCMaeeiAaGgabeaaaOqaaiabdogaJjabgkHiTiabdogaJnaaBaaaleaacqqGObaAcqqGPbqAcqqGNbWzcqqGObaAaeqaaaGcbaGaeeyAaKMaeeOzayMaeeiiaaIaem4yamMaeyOpa4Jaem4yam2aaSbaaSqaaiabbIgaOjabbMgaPjabbEgaNjabbIgaObqabaaaaaGccaGL7baaaaa@724E@

Here *c *is the (logarithmic) active concentration of the metabolite. The penalty function for the whole concentration vector is

s(C)=∑c component of Cs(c).
 MathType@MTEF@5@5@+=feaafiart1ev1aaatCvAUfKttLearuWrP9MDH5MBPbIqV92AaeXatLxBI9gBaebbnrfifHhDYfgasaacH8akY=wiFfYdH8Gipec8Eeeu0xXdbba9frFj0=OqFfea0dXdd9vqai=hGuQ8kuc9pgc9s8qqaq=dirpe0xb9q8qiLsFr0=vr0=vr0dc8meaabaqaciaacaGaaeqabaqabeGadaaakeaacqWGZbWCcqGGOaakcqqGdbWqcqGGPaqkcqGH9aqpdaaeqbqaaiabdohaZjabcIcaOiabdogaJjabcMcaPaWcbaGaem4yamMaeeiiaaIaee4yamMaee4Ba8MaeeyBa0MaeeiCaaNaee4Ba8MaeeOBa4MaeeyzauMaeeOBa4MaeeiDaqNaeeiiaaIaee4Ba8MaeeOzayMaeeiiaaIaee4qameabeqdcqGHris5aOGaeiOla4caaa@4D2F@

**Figure 1:**
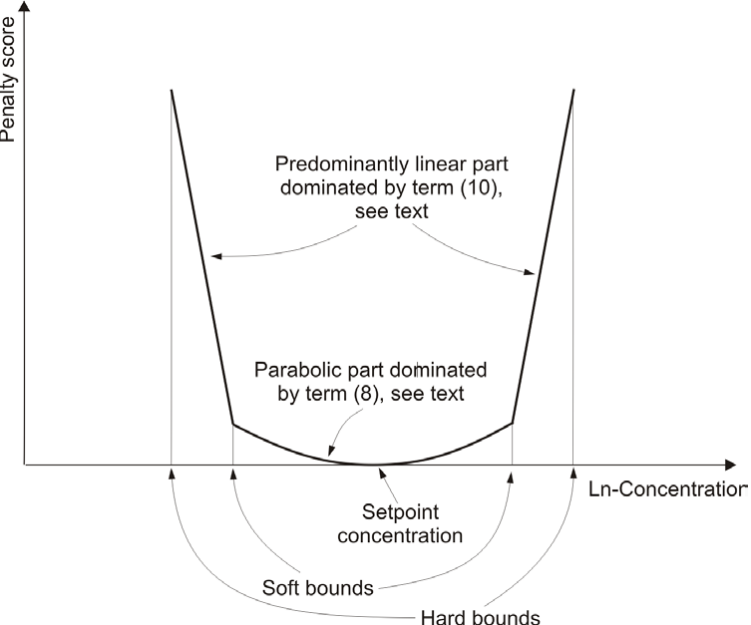
**Penalty score for concentration values**. Illustration of the penalty score used in the objective function to incorporate constraints to metabolite concentrations. The x-axis refers to logarithmic concentration values of the metabolite. If the concentration value coincides with the set-point value the penalty score is zero. For concentration values lying within the soft bounds the score depends quadratically on the distance from the set-point value. For concentration values lying outside the soft bounds the score increases linearly with increasing distance from the adjacent soft bound. Concentration values outside the hard bounds are forbidden as indicated by the truncated line.

As in Henry et al. [23] we introduce a second type of bounds, so-called hard bounds, to exclude metabolite concentrations which are impossible from the biochemical point of view. The combined effect of set-point values, soft and hard concentration bounds on the scoring function of the optimization algorithm is shown in figure 1.

Metabolic network models may contain reactions which are simplified in a way that reactants are dropped from the reaction formula. For example, the oxidation of glutathione (GSH) to glutathione disulfide (GSSG) is usually written as an overall reaction 2GSH → GSSG. Actually, this reaction should read 2GSH + R-OOH → GSSG + R-OHH_2_O where R-OOH represents a large group of not further specified hydroperoxides that can be detoxified by the glutathione system. For these lumped reactions it is impossible to give a realistic ΔGr0
 MathType@MTEF@5@5@+=feaafiart1ev1aaatCvAUfKttLearuWrP9MDH5MBPbIqV92AaeXatLxBI9gBaebbnrfifHhDYfgasaacH8akY=wiFfYdH8Gipec8Eeeu0xXdbba9frFj0=OqFfea0dXdd9vqai=hGuQ8kuc9pgc9s8qqaq=dirpe0xb9q8qiLsFr0=vr0=vr0dc8meaabaqaciaacaGaaeqabaqabeGadaaakeaacqqHuoarcqqGhbWrdaqhaaWcbaGaeeOCaihabaGaeeimaadaaaaa@31A6@ value. For other reactions a ΔGr0
 MathType@MTEF@5@5@+=feaafiart1ev1aaatCvAUfKttLearuWrP9MDH5MBPbIqV92AaeXatLxBI9gBaebbnrfifHhDYfgasaacH8akY=wiFfYdH8Gipec8Eeeu0xXdbba9frFj0=OqFfea0dXdd9vqai=hGuQ8kuc9pgc9s8qqaq=dirpe0xb9q8qiLsFr0=vr0=vr0dc8meaabaqaciaacaGaaeqabaqabeGadaaakeaacqqHuoarcqqGhbWrdaqhaaWcbaGaeeOCaihabaGaeeimaadaaaaa@31A6@ value is simply not known (e. g. for 37 reactions in *E. coli *[23]). For such reactions the consensus rule (eq. 1) is not applied.

#### Setting up the constrained optimization problem

In FBA, formulation of the optimization problem requires to define the following three elements: (i) a physiologically meaningful scoring function to evaluate flux distributions, (ii) the steady-state conditions for all internal metabolites valid for the time-scale of interest (e. g. the time-scale of growth) and (iii) further constraints taking into account biochemical knowledge as, for example, maximal enzyme capacities limiting the flux rates [10] or thermodynamic constraints on flux directions as those discussed above. The steady-state condition can be formulated as

**S'**V = 0

where **S' **derives from the full stoichiometric matrix **S **of the network upon deletion of all columns referring to those metabolites which are exchanged with the external environment and thus need not to be balanced. According to the principle of minimal fluxes [8,9] we set up the scoring function as the sum of the absolute values of all reaction fluxes |V| while assigning fixed values *L*_*j*_, *j *∈ *J *to all output fluxes, which are directly linked to cellular functions, the so-called target fluxes, the set of which is denoted by *J*. Adding the weighted terms (eq. 6) and (eq. 8) to the scoring function and including the constraint (eq. 9) the complete optimization problem is written as

Minimize|V|+λ1s(C)+λ2∑m∈W(cm−w(m))2SubjecttoS′V=00≤vj+αdj≤α,1≤j≤n0≤−ΔGrj+αdj≤α,1≤j≤nΔGr¯/(RT)=ΔGr0¯/(RT)+SCC∈ℭvj=Lj,j∈J.
 MathType@MTEF@5@5@+=feaafiart1ev1aaatCvAUfKttLearuWrP9MDH5MBPbIqV92AaeXatLxBI9gBaebbnrfifHhDYfgasaacH8akY=wiFfYdH8Gipec8Eeeu0xXdbba9frFj0=OqFfea0dXdd9vqai=hGuQ8kuc9pgc9s8qqaq=dirpe0xb9q8qiLsFr0=vr0=vr0dc8meaabaqaciaacaGaaeqabaqabeGadaaakeaafaqaaeWbcaaaaeaaieqacqWFnbqtcqWFPbqAcqWFUbGBcqWFPbqAcqWFTbqBcqWFPbqAcqWF6bGEcqWFLbqzaeaadaabdaqaaiabbAfawbGaay5bSlaawIa7aiabgUcaRGGaciab+T7aSnaaBaaaleaacqaIXaqmaeqaaOGaem4CamNaeiikaGIaee4qamKaeiykaKIaey4kaSIae43UdW2aaSbaaSqaaiabikdaYaqabaGcdaaeqbqaaiabcIcaOiabdogaJnaaBaaaleaacqWGTbqBaeqaaOGaeyOeI0Iaem4DaCNaeiikaGIaemyBa0MaeiykaKIaeiykaKYaaWbaaSqabeaacqaIYaGmaaaabaGaemyBa0MaeyicI4Saee4vaCfabeqdcqGHris5aaGcbaGae83uamLae8xDauNae8NyaiMae8NAaOMae8xzauMae83yamMae8hDaqNae8hiaaIae8hDaqNae83Ba8gabaGaf83uamLbauaacqqGwbGvcqGH9aqpcqaIWaamaeaaaeaafaqabeqacaaabaGaeGimaaJaeyizImQaemODay3aaSbaaSqaaiabdQgaQbqabaGccqGHRaWkcqGFXoqycqWGKbazdaWgaaWcbaGaemOAaOgabeaakiabgsMiJkab+f7aHjabcYcaSaqaaiabigdaXiabgsMiJkabdQgaQjabgsMiJkabd6gaUbaaaeaaaeaafaqabeqacaaabaGaeGimaaJaeyizImQaeyOeI0IaeuiLdqKaee4raC0aa0baaSqaaiabbkhaYbqaaiabdQgaQbaakiabgUcaRiab+f7aHjabdsgaKnaaBaaaleaacqWGQbGAaeqaaOGaeyizImQae4xSdeMaeiilaWcabaGaeGymaeJaeyizImQaemOAaOMaeyizImQaemOBa4gaaaqaaaqaamaanaaabaGaeuiLdqKaee4raC0aaSbaaSqaaiabbkhaYbqabaaaaOGaei4la8IaeiikaGIaeeOuaiLaemivaqLaeiykaKIaeyypa0Zaa0aaaeaacqqHuoarcqqGhbWrdaqhaaWcbaGaeeOCaihabaGaeGimaadaaaaakiabc+caViabcIcaOiabbkfasjabdsfaujabcMcaPiabgUcaRiab=nfatjabboeadbqaaaqaaiabboeadjabgIGioprr1ngBPrMrYf2A0vNCaeHbfv3ySLgzGyKCHTgD1jhaiqaacqqFTeYqaeaaaeaafaqabeqacaaabaGaemODay3aaSbaaSqaaiabdQgaQbqabaGccqGH9aqpcqWGmbatdaWgaaWcbaGaemOAaOgabeaakiabcYcaSaqaaiabdQgaQjabgIGiolabdQeakjabc6caUaaaaaaaaa@CC8B@

ℭ
 MathType@MTEF@5@5@+=feaafiart1ev1aaatCvAUfeBSjuyZL2yd9gzLbvyNv2Caerbhv2BYDwAHbqedmvETj2BSbqee0evGueE0jxyaibaiKI8=vI8tuQ8FMI8Gi=hEeeu0xXdbba9frFj0=OqFfea0dXdd9vqai=hGuQ8kuc9pgc9s8qqaq=dirpe0xb9q8qiLsFr0=vr0=vr0dc8meaabaqaciGacaGaaeqabaqadeqadaaakeaatuuDJXwAKzKCHTgD1jharyqr1ngBPrgigjxyRrxDYbaceaGae8xlHmeaaa@3F03@ is a vector of ranges defined by the hard concentration bounds. V is the vector of flux rates and *v*_*j *_is the *j*-th component of V. *λ*_1_, *λ*_2 _∈ ℝ^+ ^are empirical factors weighting the relative contribution of the various penalty scores relative to the scoring function of fluxes. (For our computations we have chosen *λ*_1 _= 100, *λ*_2 _= 0.01 putting a lower weight to the attainment of set-point concentration values than to the restriciton of the metaboite concentration values to physiologically feasible soft bounds.) *n *is the number of reactions, and for any 1 ≤ *j *≤ *n*, *d*_*j *_is a binary variable. *α *is set to a positive number which is larger than any possible flux value and larger than any possible Gibb's energy value, and it can easily be shown that the constraints 0 ≤ *v*_*j *_+ *αd*_*j *_≤ *α *and 0 ≤ -ΔGrj
 MathType@MTEF@5@5@+=feaafiart1ev1aaatCvAUfKttLearuWrP9MDH5MBPbIqV92AaeXatLxBI9gBaebbnrfifHhDYfgasaacH8akY=wiFfYdH8Gipec8Eeeu0xXdbba9frFj0=OqFfea0dXdd9vqai=hGuQ8kuc9pgc9s8qqaq=dirpe0xb9q8qiLsFr0=vr0=vr0dc8meaabaqaciaacaGaaeqabaqabeGadaaakeaacqqHuoarcqqGhbWrdaqhaaWcbaGaeeOCaihabaGaemOAaOgaaaaa@321C@ + *αd*_*j *_≤ *α *are equivalent to *v*_*j *_≠ 0 → sgn(*v*_*j*_) = -sgn (ΔGrj
 MathType@MTEF@5@5@+=feaafiart1ev1aaatCvAUfKttLearuWrP9MDH5MBPbIqV92AaeXatLxBI9gBaebbnrfifHhDYfgasaacH8akY=wiFfYdH8Gipec8Eeeu0xXdbba9frFj0=OqFfea0dXdd9vqai=hGuQ8kuc9pgc9s8qqaq=dirpe0xb9q8qiLsFr0=vr0=vr0dc8meaabaqaciaacaGaaeqabaqabeGadaaakeaacqqHuoarcqqGhbWrdaqhaaWcbaGaeeOCaihabaGaemOAaOgaaaaa@321C@). Intentionally, for a zero flux through a reaction the change of Gibb's free energy is not constrained because it might be substantially different from zero if the corresponding enzyme is missing or inhibited. The optimization problem corresponds to a mixed integer (boolean) linear program with quadratic scoring function.

We call a flux distribution obtained by solving the above optimization problem (eq. 10) thermodynamically realizable and refer to it in the following as **TR-fluxmin**, i.e. **t**hermodynamically **r**ealizable **flux**-**min**imized solution. If the maximization of biomass is used as flux objective, the sum of internal fluxes appearing in the objective function is replaced by the negative biomass production rate.

### Testing

#### Application to a metabolic network of human erythrocytes

**Figure 2:**
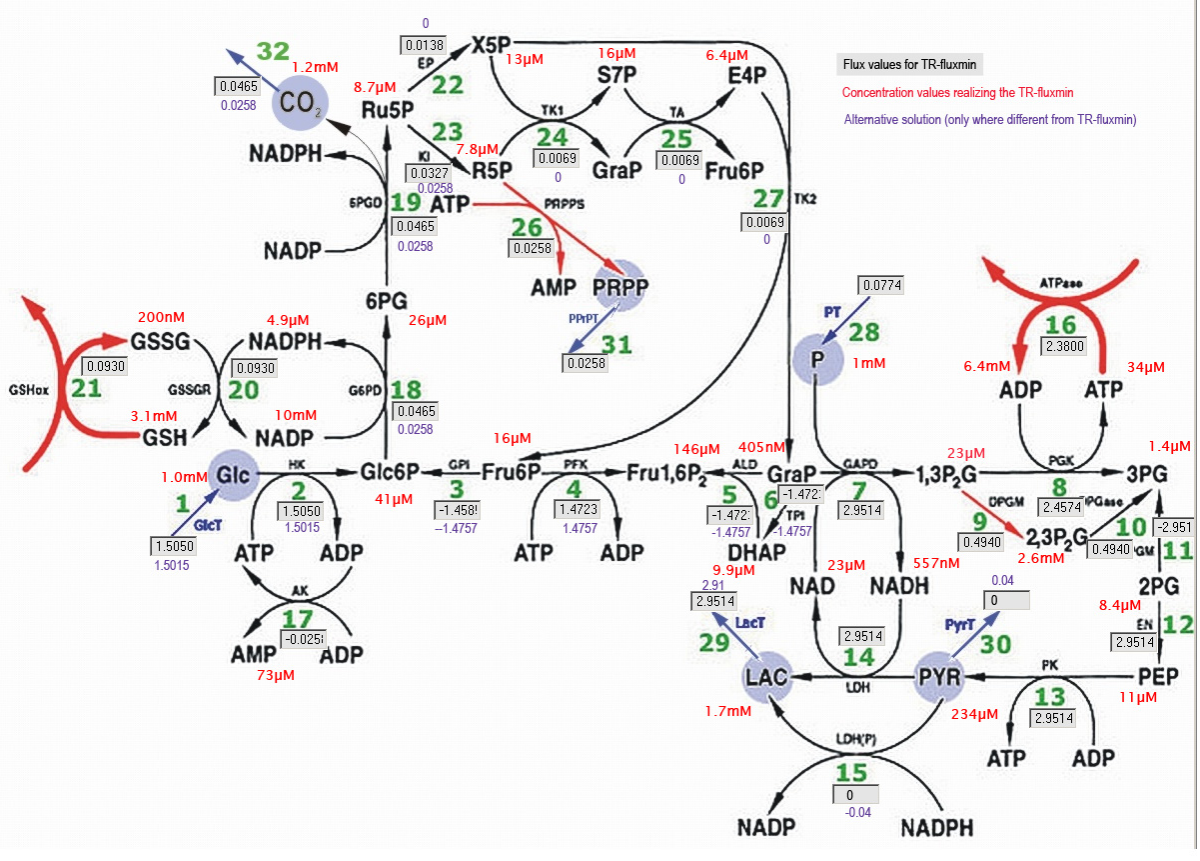
**Reaction scheme of the human erythrocyte network**. The flux values of the TR-fluxmin solution are marked in the boxes with grey background (fluxes in mmol/h). The observed concentration values used as set-points are given in red near the metabolite. Flux values of the first alternative TR-fluxmin solution that appeared in the perturbation analyses are written in violet near to the boxes if they differ from the value inside the box. The scheme has been generated with the aid of the program 'FluxAnalyzer' [44].

The method described above was applied to a metabolic network of human red blood cells [24,33] for which stationary flux distributions have already been calculated in our previous work [8]. The network comprises basically two cardinal metabolic pathways of this cell: glycolysis including the so-called 2,3-bisphosphoglycerate shunt, and the pentose phosphate cycle dividing into an oxidative and a non-oxidative part. The network consists of 27 biochemical reactions, 5 transport processes and 32 metabolites (see figure 2 and the supplementary material for the complete description of the model). The orientation of the arrows in the reaction scheme corresponds to the net direction of the reaction flux at standard concentrations. Standard Gibb's energies have been derived from the equilibrium constants contained in the kinetic model [24,33]. The functionally essential target reactions that have to be maintained by the network are the following: (i) formation of 2,3-bisphospho-D-glycerate (2,3P_2_G, reaction #9) required to modulate oxygen affinity of hemoglobin, (ii) ATP-utilization (ATPase, #16), which is mostly spent on the Na^+^/K^+^-ATPase to build up the Na^+^/K^+^-gradient across the plasma membrane, (iii) oxidation of GSH (GSHox, #21) to prevent oxidative damage of cellular proteins and lipids, (iv) synthesis of PRPP (PRPPS, #26) required for the salvage of adenine nucleotides. The magnitude of these 4 target reactions depends on the specific external conditions of the cell as, for example, osmolarity of the blood or preservation medium, oxidative stress caused by reactive oxygen species, or lowering of the oxygen tension during hypoxia. In our calculations the flux values for these 4 target reactions were chosen as reported for the normal in vivo state of erythrocytes: DPGM = 0.49 mmol/h, ATPase = 2.38 mmol/h, GSHox = 93 *μ*mol/h, PRPPS = 26 *μ*mol/h. With these values for the target fluxes, the comprehensive kinetic model [24,33] yielded metabolite concentrations as shown in figure 2. These values are in good concordance with experimentally determined concentrations and thus will be referred to in the following as 'observed' concentrations.

Using the same values of standard Gibb's free reaction energy changes as used in the kinetic model and putting the set-point values of the metabolite concentrations to the 'observed' ones, the TR-fluxmin solution of the optimization problem turns out to be identical with the flux-minimized solution determined by our previous approach [8]. A detailed description of the model and the solution mentioned below can be found in the supplements [see Additional file 1].

##### Perturbation analysis

**Figure 3:**
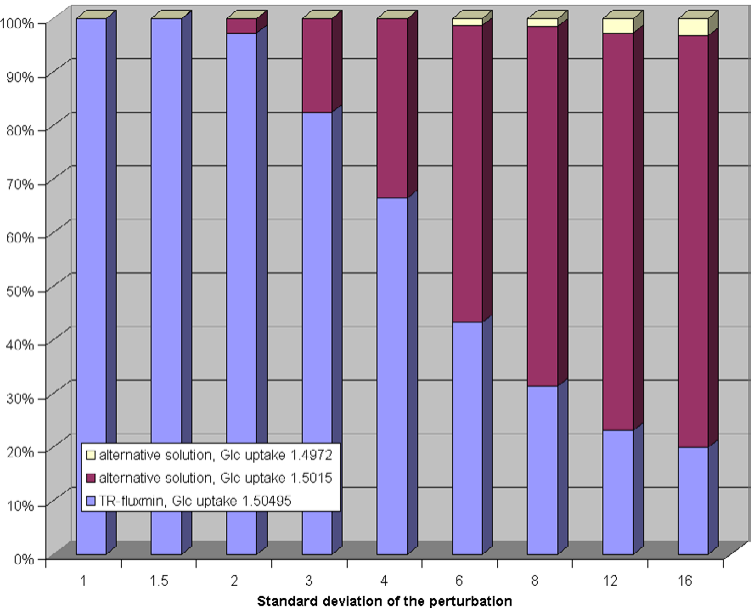
**Impact of random perturbations of observed metabolite concentrations on calculated flux distributions for the erythrocyte network**. Each bar shows the relative frequency of alternative flux distributions (y-axis) in dependence on the standard deviation of the perturbation (x-axis) of the set-point concentration values. The perturbationanalysis is based on random numbers generated with the Cliff random number generator [45] transformed to normal distribution by theBox-Muller method [46] where the expectation value is fixed to zero and for each series (1000 runs) the standard deviation is fixed to a specified value (given in the figures). For the perturbation of the concentrations each concentration value is multiplied with *e*^*X *^where *X *is a newly generated pseudo-random number. Each concentration value is modified independently from the perturbation of the other concentration values. The standard deviation value *s *can be translated to a 'deviation factor' as *e*^*s*^. For the perturbation of the equilibrium constants each value is also multiplied with *e*^*X *^where *X *is a newly generated pseudo-random number. After the perturbation the values are modified again to ensure well-formedness by the algorithm described in the text. The standard deviation value *s *can be translated into deviations of Gibb's free energy change as 2.58·*s *kJ/mol.

To investigate the impact of errors in the observed metabolite concentrations on the predicted flux distribution the concentration values given in figure 2 were perturbed by multiplying them with a random factor obeying an exponential normal distribution with controlled standard deviation (see caption of figure 3). The hard concentration bounds were chosen as follows: 0.1 *μ*M...100 mM (glucose), 0.1 *μ*M...25 mM (CO_2 _and phosphate), and 0.01 *μ*M...10 mM (28 remaining compounds). Calculation of the flux distribution with randomly altered set-point concentration values was repeated in 1000 trials. Surprisingly, for smaller perturbations the reference solution (= TR-fluxmin for 'true' set-point values) was retained in all trials. But perturbations with a standard deviation of 2 (corresponding to an average factor 7.4 in the change of the concentration values) resulted in a second, slightly different, flux distribution in some trials. For perturbations with standard deviation of 6 (corresponding to an average 400-fold change in the concentration values) this second alternative flux distribution dominated and on top a third alternative flux distributions was obtained in a significant number of trials (see figure 3).

**Figure 4:**
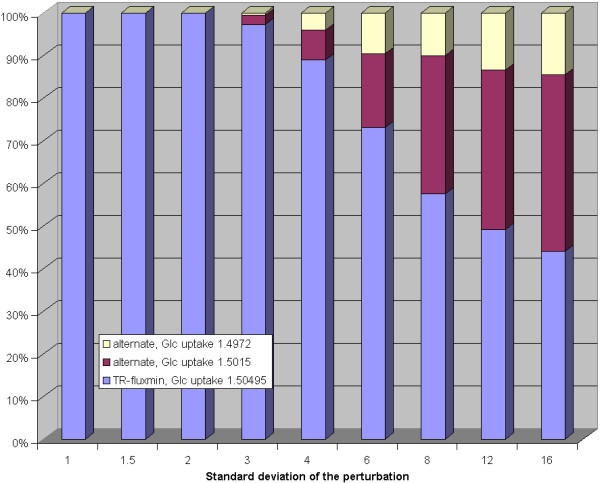
**Impact of random perturbations of equilibrium constants on calculated flux distributions for the erythrocyte network**. Each bar shows the relative frequency of alternative flux distributions (y-axis) in dependence on the standard deviation of the perturbation (x-axis) of the equilibrium constants.

In a second perturbation analysis, the standard Gibb's energy change values were randomly altered in a similar manner. Also here, there was an increasing tendency towards the two alternative flux distributions found before when increasing the magnitude of perturbations (see figure 4). These alternative flux distributions already occurred at a standard deviation of 3 (corresponding to an average deviation of 7.7 kJ/mol of the standard Gibb's energy changes) and their relative share became dominant at a standard deviation value of 6 (corresponding to an average deviation of 15.5 kJ/mol of the standard Gibb's energy changes).

##### Inspection of alternative flux distributions

The three alternative flux distributions obtained in the perturbation studies differ in the uptake flux of glucose (1.50495 mmol/h for the unperturbed network; 1.5015 mmol/h and 1.4972 mmol/h for the two alternative flux distributions) and the fluxes in the non-oxidative pentose phosphate pathway converting ribulose-5-phosphate (Ru5P) into fructose-5-phosphate (Fru6P) and glyceraldehyde 3-phosphate (GraP). The reference solution predicts this pathway to proceed in forward direction thereby forming 20.7 *μ*mol/h ribulose-5-phosphate. In the second solution (glucose uptake 1.5015 mmol/h) this pathway is not used at all whereas in the third flux distribution (glucose uptake 1.4972 mmol/h) it is used in backward direction producing 25.8 *μ*mol/h ribulose-5-phosphate. Interestingly, the latter flux distribution is also obtained for the unperturbed network if the maximization of biomass production used as flux criterion. Notably, all three different flux distributions obtained as solution of the minimization problem (eq. 10) for randomly altered thermodynamic parameters and set-point concentrations are feasible from the kinetic view point, i.e. the kinetic model of the erythrocyte metabolism yields a stable stationary solution.

##### Effect of external concentrations

Our algorithm allows assessing how the predicted flux distributions are affected by changes in the concentration of external metabolites. In vivo, such a situation may occur if some essential fuels for the cellular metabolism are depleted, for example, due to a reduced blood flow through vessels with severe atherosclerotic stenoses, or some end products of the cellular metabolism accumulate because of a reduced excretion capacity of the body. For example, in case of strong physical exercise the concentration of lactate in human blood may rise to values as high as 19.5 mM (in blood plasma) respectively 7.0 mM (in erythrocyte cytoplasm) [34] indicating that the lactate production by the anaerobic skeletal muscle clearly exceeds its rate of re-conversion to glucose in the liver and its utilization rate in the heart muscle. To investigate the consequences of such high blood lactate levels for the metabolism of red cells we calculated thermodynamically realizable flux distributions at gradually increasing concentration of external lactate. For all metabolites except external lactate, the hard bounds were put to ± 25% and the soft bounds to ± 10% deviation from of the 'observed' concentration values. For external lactate concentrations up to a critical value of 12.4 mM our algorithm predicted a thermodynamically realizable flux distribution. For concentrations higher than 12.4 mM no stationary flux distribution solution was found. Increasing gradually the concentration of external lactate up to the critical value of 12.4 mM, the concentrations of pyruvate, NAD^+ ^and NADH tended towards the hard bounds to ensure the flux through the lactate dehydrogenase (EC:1.1.1.27) to be directed towards formation of lactate. Our find of a metabolic threshold effect with respect to blood lactate levels corresponds well with clinical observations. At lactate levels higher than 4 mM a reduced deformability of erythrocytes is observed, which may account for the exercise-induced arterial hypoxemia occuring in athletes [35]. Decreasing deformability of erythrocytes is a clear indication for a severely perturbed metabolism of the cell.

#### Application to a metabolic network of *E. coli*

To check the applicability of our algorithm to genome-scale metabolic networks comprising hundreds of reactions and metabolites, we performed the same type of analysis as described above with respect to the metabolic network iJR904 of the bacterium *E. coli *reconstructed by Palsson and co-workers [25]. In this model a minimal medium composed of glucose, ammonium, sulfate, oxygen, phosphate is sufficient for growth according to the biomass creation formula associated with the model. Experimental flux data for *E. coli *has been determined by Emmerling et al. [36] which correspond to 17 internal fluxes of the iJR904 network (using the projection of Segre et al. [37] onto the iJE660a network of *E. coli *[38].) The thermodynamic properties of the iJR904 network, consisting of 659 metabolites and 931 reactions, have been analyzed previously [14,15,20,23,32]. Since experimentally determined Gibb's free energies are available only for a minor fraction of reactions [20,39] we use computed values given by Henry et al. [23].

These values were obtained by a slightly modified version of the group contribution method [40,41]. Physiological concentration ranges were available for 22 internal metabolites (given in Kümmel et al. [14]) and 10 external metabolites (given in Henry et al. [23]). For the other metabolites generic concentration bounds were used based on typical cellular concentration ranges reported in the literature: 20 *μ*M-0.5 mM (soft bounds), 5 *μ*M-2 mM (hard bounds). Further details of the model are given in the supplement [see Additional file 2].

**Figure 5:**
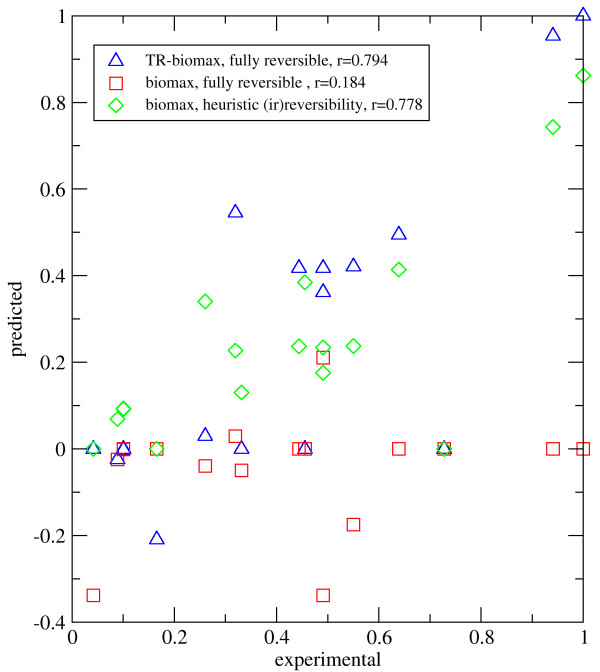
**Comparison between measured and predicted flux rates of E. coli**. x-axis: observed flux rates measured by Emmerling et al. [36]. y-axis: predicted flux rates obtained with three different variants of FBA. Fluxes were transformed into dimensionless units by putting the highest observed and calculated value equal to unity. All flux distributions were obtained by using the maximization of biomass as flux objective. Blue triangles: All reactions are fully reversible (except exchange fluxes and 37 internal fluxes with unknown value of free energy changes); solution of the complete optimization problem (eq. 10), i.e. condition of thermodynamic realizability included. Red squares: All reactions are fully reversible (except the exchange fluxes and 37 internal fluxes with unknown value of free energy changes); solution to biomass maximization at fixed glucose intake; the condition of thermodynamic realizability was omitted. Green diamonds: Irreversibility of 532 reactions prescribed as originally proposed by Reed et al. [25]; solution to biomass maximization at fixed glucose intake. Coefficients marked with *r *= denote the Pearson's product-moment correlation coefficient.

We calculated the flux distribution in this network according to the proposed optimization principle (eq. 10) using as flux objective the maximization of the biomass production. No a priori assumptions were made with respect to the directionality of reactions with two exceptions: The direction of the exchange fluxes was fixed according to the experimental conditions [36] and the direction of 37 internal reactions for which no Gibb's energy value was given in Henry et al. [23] was also fixed [see Additional file 2, archive member 'Ecoli-model.txt', section 'reactions excluded from the TR-property', to see which]. As shown in Fig. 5) (case: 'TR-biomax', data points symbolized by blue triangles) the thermodynamically realizable solution provided a good concordance with observed flux values available for 17 internal reactions. To check the influence of the thermodynamic side constraints on the quality of the flux distribution, we omitted the condition of thermodynamic realizability from the optimization algorithm, again making no a priori assumptions on flux directionalities. In this case ('biomax, fully reversible', data symbolized by red squares in Fig. 5)) the concordance between predicted and observed flux values diminished significantly. This example shows that our algorithm may significantly improve the reliability of flux predictions even if the concentration range of most metabolites is only roughly restrained.

In a third calculation we again omitted the condition of thermodynamic realizability from the optimization algorithm but instead used the heuristic classification of reactions into reversible and irreversible ones as outlined in [25] (case: 'biomass, heuristic irreversibilities', data points symbolized by green diamonds). The obtained flux distribution also yielded a reasonably good concordance between predicted and observed flux values. Notably, this 'classical' variant of FBA gave no better predictions of the observed fluxes than the TR-solution obtained with our algorithm. This qualifies our method as a valuable flux predictor for large-scale networks without the need to apply heuristic rules for the assignment of flux directionalities.

##### Perturbation analysis

Using the same perturbation analysis as outlined above for the erythrocyte network we investigated the impact of alterations in the values of the Gibb's free standard energies on the predicted flux distributions. Such an analysis is of importance as the values of standard Gibb's free energy changes computed by the group contribution method may generally exhibit a large degree of uncertainty [42].

**Figure 6:**
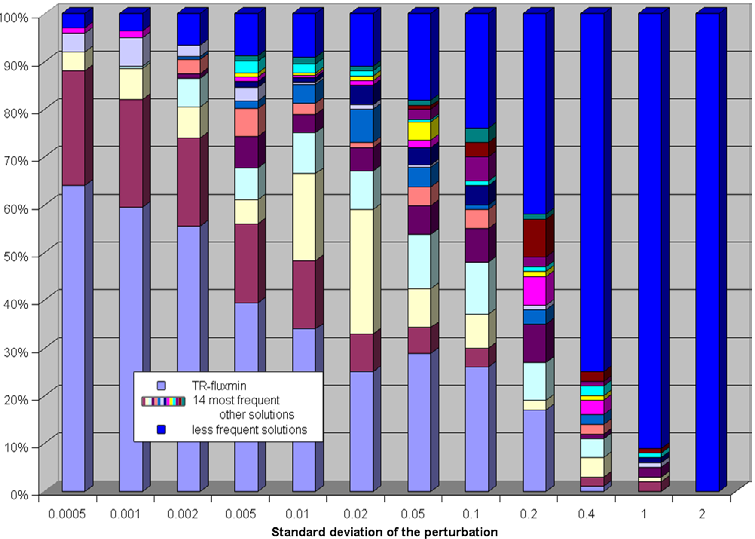
**Equilibrium constants perturbation analysis for the E. coli network**. Each bar shows the relative frequency of the 15 most frequently occurring alternative flux distributions (y-axis) in dependence on the standard deviation of the perturbation (x-axis) of the equilibrium constants. The less frequently occurring solutions are summarized in the bar designated by 'rest'.

Compared with the findings for the erythrocyte network, much smaller perturbations already resulted in a multitude of alternative flux distributions (see figure 6). Thus, the higher the complexity of the network the more susceptible is the predicted flux distribution is to the choice of the standard Gibb's energies. Closer inspection of the predicted alternative flux distributions showed that the main differences are concentrated in some distinct parts of the networks. We found the largest variability of predicted fluxes for the exchange of CO_2_, the 3-reaction pathway leading from acetaldehyde and CoA to formation of ATP from ADP via acetyl-phosphate as intermediate, and in the import of *a*-ketoglutarate. The possible fluxes through these reactions appear to be strongly determined by thermodynamic constraints and thus are difficult to predict given low accuracy of thermodynamic data.

## Discussion & Conclusion

Quantitative evaluation of genome-scale metabolic models by means of FBA is becoming more and more appealing because it works without knowledge of the kinetics and regulation of the underlying enzymes and membrane transporters. However, the outcome of FBA is rather hypothetical because it relies on plausible but hardly provable optimality principles that are thought to govern metabolic flux distributions.

Therefore, a challenge for computational systems biology lies in the incorporation of all biochemical knowledge that is obtainable at genome-scale (which is not the case for enzyme kinetics). One important restriction of fluxes in the network arises from thermodynamics. Reactions associated with a decrease of free energy larger than 30 kJ/mol are generally thought to be irreversible. This condition can be used as an additional constraint on feasible flux distributions. Kümmel and co-workers [20] have recently developed an algorithm that – based on thermodynamics, network topology and heuristic rules – automatically assigns reaction directions in metabolic models such that the reaction network is thermodynamically feasible with respect to the production of energy equivalents. However, an a priori distinction between reversible and irreversible reactions may become problematic under extreme conditions, e.g. depletion of substrates or accumulation of intermediates due to inhibition of enzymes, where metabolite concentrations may drastically change thus allowing to reverse reactions that are normally designated to be irreversible.

For example, under hypoxic conditions, the cellular concentration of oxygen may become so low that the respiratory chain – usually thought to carry electrons from hydrogen to oxygen in a strictly irreversible manner – may indeed operate in the reverse direction, i.e. reducing NAD to NADH_2 _[43]. Thus, it is necessary to replace the rigid priori classification of reactions into reversible and irreversible ones by a more flexible constraint that assures the flux directions to be compatible with the change of Gibb's free energies, exhibiting a wide range of values depending on the actual metabolite concentrations. An important step into this direction was recently made by Henry et al. [23] who included the thermodynamic consensus rule as additional side constraint into FBA. In their study, they investigated the range of metabolite concentrations that allow in a genome-scale network of *E. coli *the realization of a specific flux distribution assuring optimal bacterial growth. In contrast to this approach, the algorithm proposed in this work aims at employing reliable information on metabolite concentrations to restrain the solution space of FBA.

Hence, depending on reported ranges of metabolite concentrations, our algorithm may yield different flux distributions. In other words, in our approach we do not ask for metabolite concentrations that are compatible with a given flux distribution but in contrast ask for the flux distribution that is compatible with a given metabolite profile. As demonstrated for two exemplary metabolic networks, even if no measurements of metabolite concentrations are available restriction of concentrations to physiologically feasible ranges alone allows the prediction of reliable flux distributions if no a priori assumptions are made on the reversibility of the reactions. As demonstrated for the erythrocyte network, our approach may provide valuable information about alterations in the external conditions of a cell that may result in a metabolic dysfunction. Of course, FBA cannot assess whether a stable steady state may exist at very high concentrations of external lactate because this is determined by kinetic regulation. Possibly the metabolite concentrations may vary even in a larger interval than imposed in our calculations. This problem can be addressed better by a comprehensive kinetic network model. Nevertheless, our method may provide valuable information on external conditions causing metabolic problems merely for thermodynamic reasons. Concerning the predictive capacity of our method it must be critically noted that – based on a comparison with a relatively small number of measured fluxes – the most reliable flux distribution for the *E. coli *network is still obtained if the directionality of fluxes is a priori defined based on biochemical conventions. This is obviously due to the fact that – owing to the lack of reliable experimental data – the soft bounds for the metabolite concentration used in our method have been too generously chosen. Indeed, enlarging systematically the physiologically feasible concentration ranges one eventually obtains a network without any constraint of flux directionalities. Hence, the usefulness of the proposed method essentially depends upon the availability of reliable information on values of free energy changes and metabolite concentrations. As long as this information is not available, the benefit of our method consists mostly in the generation of alternative flux distributions by varying the values of standard Gibb's free energy changes and/or in the physiologically relevant concentration ranges of metabolites. Applying such perturbation analysis to two networks of different complexity has provided evidence that the larger the network is, the more alternative flux distributions occur, even at relatively modest variation of energy values of about 5 kJ/mol. Inspection of such alternative flux distributions reveals critical reactions for which fluxes are largely undetermined by the FBA approach. In this respect, our method represents a useful complement to the thermodynamic evaluation method recently proposed by Kümmel et al. to identify putative regulatory sites by network-embedded thermodynamic analysis of metabolome data [14].

## Authors' contributions

The basic idea of the presented theoretical concept was developed by HGH. AH and SH prepared of the network models. AH developed the algorithms and carried out the computations. All authors jointly contributed to the manuscript.

## Supplementary Material

Additional file 3 This document shows the proof that thermodynamic realizability implies that there is no net flux in a closed loop – a consequence of the generalization of Kirchhoff's loop law. Click here for file

Additional file 1 This archive contains three files relating to the erythrocyte network. The file "Ery-model.txt" gives the definition of the network as tab-separated text file which is organized in the sections: 'Metabolites', 'Reactions', 'Reactions excluded from the TR property', 'Equilibrium constants', 'Targetfluxes', 'Fixed concentrations', The file "Ery.sbml" gives the network description in SBML format. The file "Ery-solutions.txt" contains three solutions: the thermodynamically realizable fluxmin solution (TR-fluxmin) together with the associated metabolite concentrations and the two alternative solutions obtained in the perturbation analysis. Click here for file

Additional file 2 This archive includes two directories relating to the *E. coli *iJR904 computations: "FullyReversible" and "HeuristicIrreversibilities". Both contain the file "Ecoli-model.txt", a tab-separated text file, as the definition of the network which is organized in the sections: 'Metabolites', 'Reactions', 'Reactions excluded from the TR property', 'Equilibrium constants', 'Targetfluxes', 'Concentration bounds', and 'Setpoint concentrations'. Both directories also contain the file "Ecoli.sbml", the network description in SBML format. The file "Biomax.txt" also residing in both directories contains the solution of the biomass maximization without the constraint of thermodynamic realizability as a tab-separated text file assigning a reaction identifier with a flux value if it is not zero.. Additionally the directory "FullyReversible" contains the file "TR-Biomax.txt" as the solution to the thermodynamically realizable biomass maximization together with the hypothetic metabolite concentrations compatible with the flux directions. Click here for file
