# Supplementary material for: Including metabolite concentrations into flux balance analysis: thermodynamic realizability as a constraint on flux distributions in metabolic networks
Source: BMC Syst Biol. 2007 Jun 1;1:23. doi: 10.1186/1752-0509-1-23 (PMC1903363; doi:10.1186/1752-0509-1-23)
Supplement: Additional file 3 — This document shows the proof that thermodynamic realizability implies that there is no net flux in a closed loop – a consequence of the generalization of Kirchhoff's loop law. [file 1752-0509-1-23-S3.pdf]

# Formal proof the the concept of thermodynamic realizability implies the generealization of Kirchhoff's loop law for metabolic networks

Andreas Hoppe*[1], Sabrina Hoffmann[1]and Hermann-Georg Holzhütter*[1]

[1]Charité – University Medicine Berlin, Institute for Biochemistry, Universitätsmedizin Berlin, Monbijoustr. 2, 10117 Berlin, Germany

Email: Andreas Hoppe - andreas.hoppe@charite.de; Hermann-Georg Holzhütter - hergo@charite.de;

*Corresponding author

Changes of the standard Gibb's energies of reactions $\Delta_\mathrm{r}\mathrm{G}_0$ can be expressed through changes of the standard Gibb's energies of the formation $\Delta_f\mathrm{G}$ of their reactants [1]:

$$\overline{\Delta_\mathrm{r}\mathrm{G}_0} = \mathbf{S}\overline{\Delta_f\mathrm{G}} \qquad (1)$$

A particular set of values standard Gibb's reaction energies are called well-formed if they satisfy this property.

The following proposition relates thermodynamic realizability to the generalization of Kirchhoff's loop law [2]:

> **Proposition.** Let the standard Gibb's reaction energy changes of a metabolic system be well-formed and let a flux vector V be thermodynamically realizable. Then the flux vector V satisfies the generalization of Kirchhoff's loop law which means that for every closed loop the net flux is zero.

**Proof.** A closed loop is characterized by a set of (reaction) indices $I$ and coefficients $\nu_i, i \in I$ which satisfy $\sum_{i\in I} \nu_i \mathbf{S}_i = \overline{0}^\mathrm{T}$ where $\mathbf{S}_i$ denotes the $i$-th row of the stoichiometric matrix $\mathbf{S}$ and $\overline{0}^\mathrm{T}$ denotes a zero row vector.

For a closed loop the sum of the respective changes of standard Gibb's energies is zero, e.g.

$$\sum_{i\in I} \nu_i \Delta_\mathrm{r}\mathrm{G}_0^{(i)} = 0 \qquad (2)$$

This is an immediate consequence of the well-formedness of the Gibb's reaction energies, see formula (1).

For the actual Gibb's energy changes we conclude:

$$\sum_{i\in I}\nu_i\Delta_\mathrm{r}\mathrm{G}^{(i)} = \sum_{i\in I}\nu_i(\Delta_\mathrm{r}\mathrm{G}_0^{(i)} + \mathbf{S}_i\mathrm{C}) = \sum_{i\in I}\nu_i\Delta_\mathrm{r}\mathrm{G}_0^{(i)} + \sum_{i\in I}\nu_i\mathbf{S}_i\mathrm{C} = (\sum_{i\in I}\nu_i\mathbf{S}_i)\mathrm{C} = \overline{0}^\mathrm{T}\mathrm{C} = 0 \qquad (3)$$

It follows that the vector $(\nu_i\Delta_\mathrm{r}\mathrm{G}^{(i)})_{i\in I}$ is neither completely positive nor completely negative. So is the vector $(\nu_i v_i)_{i\in I}$, by the directionality law. This is equivalent with the statement that this loop carries no net flux. q.e.d.

## References


1. Alberty R: *Thermodynamics of Biochemical Reactions.* Hoboken, NJ: Wiley & Sons 2003.

2. Price N, Famili I, Beard D, Palsson B: **Extreme pathways and Kirchhoff's second law**. *Biophys J* 2002, **83**(5):2879–2882.
